# Contraception for adolescents in low and middle income countries: needs, barriers, and access

**DOI:** 10.1186/1742-4755-11-1

**Published:** 2014-01-02

**Authors:** Venkatraman Chandra-Mouli, Donna R McCarraher, Sharon J Phillips, Nancy E Williamson, Gwyn Hainsworth

**Affiliations:** 1 Department of Reproductive Health and Research, World Health Organization, Avenue Appia 20, 1211 Geneva 27, Switzerland; 2 FHI 360, 2224 E NC Highway 54, Durham, NC 27713 USA; 3 Maternal and Child Health Department, Gillings School of Global Public Health, University of North Carolina, 401 Rosenau Hall, CB #7445, Chapel Hill, NC 27599-2017 USA; 4 Adolescent Sexual and Reproductive Health, Pathfinder International, 9 Galen Street, Suite 217, Watertown, MA 02472 USA

**Keywords:** Adolescents, Contraception, Low and middle income countries

## Abstract

Substantial numbers of adolescents experience the negative health consequences of early, unprotected sexual activity - unintended pregnancy, unsafe abortions, pregnancy-related mortality and morbidity and Sexually Transmitted Infections including Human Immunodeficiency Virus; as well as its social and economic costs. Improving access to and use of contraceptives – including condoms - needs to be a key component of an overall strategy to preventing these problems. This paper contains a review of research evidence and programmatic experiences on needs, barriers, and approaches to access and use of contraception by adolescents in low and middle income countries (LMIC). Although the sexual activity of adolescents (ages 10–19) varies markedly for boys versus girls and by region, a significant number of adolescents are sexually active; and this increases steadily from mid-to-late adolescence. Sexually active adolescents – both married and unmarried - need contraception. All adolescents in LMIC - especially unmarried ones - face a number of barriers in obtaining contraception and in using them correctly and consistently. Effective interventions to improve access and use of contraception include enacting and implementing laws and policies requiring the provision of sexuality education and contraceptive services for adolescents; building community support for the provision of contraception to adolescents, providing sexuality education within and outside school settings, and increasing the access to and use of contraception by making health services adolescent-friendly, integrating contraceptive services with other health services, and providing contraception through a variety of outlets. Emerging data suggest mobile phones and social media are promising means of increasing contraceptive use among adolescents.

## Introduction

An estimated 16 million adolescents aged 15–19 give birth each year [1]. Complications from pregnancy and childbirth are the leading cause of death in girls aged 15-19 in Low and Middle Income Countries (LMIC) where almost all of the estimated 3 million unsafe abortions occur [2]. Perinatal deaths are significantly higher in babies born to adolescent mothers than in those born to mothers aged 20–29 years, as are other problems such as low birth weight [2]. Preventing adolescent pregnancy is a key strategy in improving maternal and infant outcomes.

This paper presents information on sexual activity and unmet need for contraception among adolescents in LMIC, barriers to access and use, and interventions that have successfully overcome these barriers.

## Methods

1. To determine the contraceptive needs of adolescents in developing countries, we disaggregated data from Demographic and Health Surveys (DHS) to explore age of sexual debut (first sexual intercourse), use of contraception, and unmet need for contraceptive services by married and unmarried adolescents. To fill gaps, we examined studies on the levels of HIV infection and unsafe abortion.

2. To identify barriers that adolescents encounter in accessing and using contraception, we drew from two systematic reviews of qualitative studies. We also drew from two papers which took stock of the field – one published in 2001 and the other in 2010.

3. To identify effective interventions to increase access and use of contraception among adolescents, we drew upon evidence gathered by the World Health Organization (WHO) to develop guidelines on preventing early pregnancy in adolescents.

### Evidence that adolescents are sexually active and have an unmet need for contraception

We analysed DHS data from 16 diverse countries having data on married and unmarried adolescents. A substantial minority of adolescents was sexually active (had had sexual intercourse) in most of these countries; and this increased steadily from mid-to-late adolescence. (Table 1) Retrospective data showed that more than a quarter of women (20–24) in Mali and Bangladesh and between 27-35% of men (20–24) in Brazil, Dominican Republic and Haiti reported that they had sex before age 15. One-quarter to three-quarters of men (20–24) in the African and the Latin American/Caribbean countries we examined, reported having sex before age 18. In 9 of the 16 countries, 40% or more of the women (20–24) reported having sex before age 18. Reported sexual activity varied widely by region, country, and sex.

**Table 1:** **Sexual activity, marriage, and childbirth for adolescents under age 15 and 18 reported by population aged 20 ~ 24 in 16 developing countries**^**1,2,3**^

**Region**	**Under 15**				**Under 18**			
	**M**	**F**	**FM**	**FB**	**M**	**F**	**FM**	**FB**
**Sub-Saharan Africa**								
Ghana, 2008	5	7	5	2	27	41	25	16
Mali, 2006	4	26	25	10	27	73	71	46
Tanzania, 2010	6	15	7	3	40	58	37	28
Zimbabwe, 2010-11	4	4	4	1	23	38	31	21
**Middle East/North Africa**								
Egypt, 2008	-	-	2	1	-	-	17	7
Jordan, 2009	-	-	1	0	-	-	10	4
Morocco, 2003-04	-	-	3	1	-	-	16	8
Yemen, 1997	-	-	14	4	-	-	48	25
**Asia/Central Asia**								
Azerbaijan, 2006	1	1	1	0	22	12	12	4
Bangladesh 2011	1	28	29	9	6	64	65	40
Cambodia, 2010	0	1	2	0	4	15	18	7
India, 2005-06	-	13	13	3	-	43	45	22
**Latin America/Caribbean**								
Brazil, 1996	33	10	4	2	75	43	24	16
Dominican Republic, 2007	27	16	14	3	72	51	40	25
Haiti, 2012	35	13	3	1	77	51	18	13
Peru, 2012	-	7	3	1	-	43	19	15

^1^**Key:** % of **M**ales (M) and **F**emales (F) 20–24 years old reporting they had intercourse by age group and % of **F**emales who reported they were **M**arried (FM) and/or gave **B**irth (FB) by age group.

^2^**Source:** The most recent DHS for each country, ICF International, 1996–2012.

^3^The symbol ‘-’ indicates no data available.

In Mali and Bangladesh, 25% and 29% respectively of women 20–24 reported that they had been married under age 15 whereas in half the countries (8/16), 7% or fewer reported marriage under age 15. An even wider range is evident for marriage under age 18: 71% of women (20–24) from Mali reported marriage under 18 in contrast to 10% in Jordan. Similar variations are apparent in the rates of births under 15 and 18. Between 0-10% of women (20–24) reported a birth before age 15 and 4-46% reported births before age 18. Not surprisingly, Table 1 shows that the percentages of adults (20–24) reporting sex, marriage, and births increase by age in every country.

Originally, never-married adolescents were excluded from the DHS and researchers had to rely on retrospective data. Recently, some countries have begun interviewing unmarried adolescents about sexual activity, use of contraception and childbearing intentions. Table 2 gives prospective estimates of current contraceptive use and unmet contraceptive need for women (15–19) for the same countries as in Table 1 except that Yemen and Brazil have been excluded due to lack of data. Unmet need includes both fecund adolescents who want to forgo childbearing or delay it for two years and are not using a method of contraception. It also includes pregnant or postpartum amenorrheic females (period not returned since last live birth in the past two years) who reported their current pregnancy was not wanted or was mistimed. In our table and in general, data are unavailable for unmarried adolescents in the Middle East/North Africa regions and the South East Asia/East Asia/Central Asia regions.

**Table 2:** **Current contraceptive use and unmet need for contraception for women aged 15–19 in 14 developing countries**^**1,2,3**^

**Region**	**Unmarried, sexually active women 15-19**^**a**^		**Currently married women 15-19**	
	**Current use**	**Unmet need**^**b**^	**Current use**	**Unmet need**^**b**^
**Sub-Saharan Africa**				
Ghana, 2008	42	53	14	62
Mali, 2006	21	63	8	35
Tanzania, 2010	40	48	15	16
Zimbabwe, 2010-2011	24	64	36	19
**Middle East/North Africa**				
Egypt, 2008	-	-	23	7
Jordan, 2009	-	-	27	8
Morocco, 2003-04	-	-	38	10
**South East Asia/East Asia/Central Asia**				
Azerbaijan, 2006	Too few	Too few	6	16
Bangladesh, 2011	Too few	Too few	47	17
Cambodia, 2010	Too few	Too few	27	16
India, 2005-06	-	-	13	27
**Latin America/Caribbean**				
Dominican Republic, 2007	41	47	46	27
Haiti, 2012	28	67	26	57
Peru, 2012	64	34	67	19

^a^Including currently unmarried female adolescents that had sex in the past 3 months.

^b^The calculation of the unmet need for family planning is based on responses to 15 questions and was recently revised. See Bradley et. al for complete definition (Bradley S, Trevor EK, Croft N, Fishel JD, Westoff CF. Revising Unmet Need for Family Planning. DHS Analytical Studies No. 25. Calverton, Maryland, USA: ICF International; 2012. Available from: http://www.measuredhs.com/pubs/pdf/AS25/AS25[12June2012].pdf).

^**1**^**Key:** % among each category.

^**2**^**Source:** The most recent DHS for each country, ICF International, 2003–2012.

^**3**^“Too few” indicates too few cases to calculate estimate; ‘-’indicates no data available.

Note: The criteria used within the Demographic and Health Surveys programme to identify women with unmet need for family planning have recently been revised (Bradley et al., 2012).

Women are considered to have unmet need for spacing if they are:

•At risk of becoming pregnant, not using contraception, and either do not want to become pregnant within the next two years, or are unsure if or when they want to become pregnant.

•Pregnant with a mistimed pregnancy.

•Postpartum amenorrheic for up to two years following a mistimed birth and not using contraception.

Women are considered to have unmet need for limiting if they are:

•At risk of becoming pregnant, not using contraception, and want no (more) children.

•Pregnant with an unwanted pregnancy.

•Postpartum amenorrheic for up to two years following an unwanted birth and not using contraception.

For unmarried adolescents (15–19), current contraceptive use ranges from 21%-64%; for the married, the range is even wider, 6%-67%. Percentages having unmet need range from 34%-67% for the unmarried and 7%-62% for the married. Unmet need is higher for the *unmarried* than the married in six out of seven countries having relevant data. This is possibly because contraceptive services are directed towards married women.

The lack of access to contraception leads to early unwanted pregnancies with tragic consequences in LMIC.

•An estimated 16 million adolescents (15–19) give birth every year, 95% in LMIC. Complications from pregnancy and childbirth are the leading cause of death for women (15–19). Births to girls under age 15 pose especially high health risks for mother and infants [2].

•Some adolescents with unintended and unwanted pregnancies choose abortion. Where access to abortion is legally or logistically restricted, most abortions are unsafe [3]. Worldwide, adolescents aged 15–19 had an estimated 3.2 million unsafe abortions in 2008.

In summary, sexual activity and unmet need for contraception, are common among adolescents with clear differences by age, sex, region and marital status. Because married adolescents are often pressured to bear children, increasing access alone will be insufficient to ensure contraceptive use. Unmarried adolescents have an unacknowledged and frequently unmeasured need for contraception. All sexually active adolescents, regardless of marital status, deserve to have their contraceptive needs acknowledged, measured, and responded to.

### Evidence of the barriers that adolescents face in obtaining and using contraception

Two systematic reviews of qualitative research studied barriers to modern contraceptive use among adolescents in LMIC [4,5]. One found seven studies that met the inclusion and quality assessment criteria - six from sub-Saharan Africa and one from South-East Asia [4]. The larger review of sexual behaviour included 268 studies (121 were high quality or contained empirical data), of which only 54 were from LMIC (not all included data on contraceptive use) [5]. In the end, the two reviews retained only a small number of studies conducted in a few countries. Both reviews concluded that the barriers that adolescents face in obtaining and using contraception are common across developing country settings and cultures.

One set of barriers is in obtaining contraceptive methods. Adolescents experience many of the same barriers that adults do, but some are specific to them. In many poor communities of LMIC, contraceptives methods are not available to adults or to adolescents [6,7]. Even when contraceptive methods are available, laws and policies prevent their provision to unmarried adolescents or to those under a certain age [6,7]. Even where there are no legal restrictions, health workers in many places refuse to provide unmarried adolescents with contraceptive information and services because they do not approve of premarital sexual activity [6,7]. And when they do provide contraceptive methods, they often limit this to condoms, wrongly believing that long acting hormonal methods and intrauterine devices are inappropriate for nulliparous women. A recently published study of public, private not-for-profit and private for-profit providers in rural Uganda confirms these barriers and points to others such as sporadic contraceptive stocks, costs and unfriendly service provision [8].

The second set of barriers is in using contraception. Even when adolescents can obtain contraception, social pressure may prevent their use. Firstly, in many places young women are under pressure to conceive and bear children soon after marriage. Contraception is considered only after a first child is born [6,7]. Secondly, the stigma surrounding contraception prevents their use by adolescents not in stable relationships. Proposing the use of a condom or carrying one can lead to a woman being considered ‘loose’ in many places [9]. Thirdly, in many places adolescents have misconceptions about the immediate and long term side effects of contraceptive methods on their health and on their future ability to bear children. Because of the resulting fears and concerns, adolescents often consider ineffective methods such as withdrawal and traditional remedies more acceptable [10]. Fourthly, because of poor understanding of how contraceptives methods work and how they should be used, adolescents use them incorrectly as is illustrated by the following statement by a young South African woman [11]: “I take a pill when I know my boyfriend is coming and we are probably going to make love. I sometimes forgot to take it before we make love so I take it after we made love.” Finally, consistent use of contraception has been shown to be problematic among adolescents. An analysis of DHS data from 40 countries revealed that in most countries adolescents are more likely to discontinue method use than older women [12]. Male condoms are the method most commonly used by adolescents given that they are readily accessible and inexpensive [7]. However, consistent condom use tends to decrease over time within stable partnerships for they are associated with being ‘unfaithful’ or as ‘not trusting’ [13]. Sporadic sex or infrequent sex is often cited as a reasons adolescents do not use methods consistently.

In summary, adolescents – especially unmarried ones – in LMIC, face a number of barriers in obtaining contraception and in using them correctly and consistently. These barriers operate at three levels – the individual, the immediate environment and the wider environment.

### Evidence on effective interventions to increase adolescents’ access to and use of contraception

In 2011, WHO issued Guidelines on preventing early pregnancy and poor reproductive outcomes in adolescents in developing countries [1]. These Guidelines were based on reviews of published systematic reviews and of individual studies, and the collective judgment of an expert panel. Increasing access to and use of contraception was one of the four outcomes to prevent early pregnancy. (The other three outcomes were preventing marriage before 18 years; increasing knowledge and understanding of the importance of pregnancy prevention; and preventing coerced sex). The studies that met the inclusion criteria for this outcome were conducted in a number of LMIC. Some focused exclusively on condom use, while others looked at hormonal contraceptives and emergency contraception (EC). Some examined the use of contraception as a primary outcome while others examined it as secondary to outcomes such as HIV prevention or changing knowledge and attitudes. Some focused on health system actions (such as over-the-counter or clinic provision of contraception) while others focused on actions directed at community leaders and members. Collectively, they demonstrated increases in contraceptive use (including condoms, hormonal contraceptives and EC) as a result of actions directed at multiple levels – laws and policies; individuals, families and communities; and health systems. The interventions discussed below are drawn from WHO’s Guidelines.

The Appendix contains a list of reviews and studies which fed into the development of WHO’s Guidelines on preventing early pregnancy and poor reproductive outcomes in adolescents in developing countries.

#### Overcoming restrictive laws and policies

In many countries, laws and policies restrict the provision of contraception to unmarried adolescents or those below a certain age. Policy makers must intervene to reform these laws and policies to ensure that adolescents are able to obtain contraceptive information, counselling and services. Policy makers should also consider providing adolescents contraception at no or reduced cost [1].

#### Making social and group norms supportive

In many societies premarital sexual activity is not considered acceptable, and there is considerable resistance to the provision of contraceptive information and services to unmarried adolescents. To overcome this barrier, it is important to improve the understanding of influential community leaders and of the community at large on adolescent’s needs for information and contraception, and the risks to their wellbeing of not responding to these needs [1].

In many places, social and group norms hinder discussion between couples about contraception. In addition, knowledge gaps and misconceptions prevent use or proper use of contraceptive methods. Mass media (radio and television programmes), peer-education, and inter-personal communication and information education communication materials (such as posters and leaflets) have been used successfully to communicate health information to adolescents, and to influence their norms [1]. In recent years, the ways adolescents communicate have changed radically. Mobile phone technology, the Internet and social media are increasingly being used even in LMIC. These technologies are potentially valuable for communicating contraceptive information and options to adolescents conveniently and discretely [14].

#### Improving knowledge and understanding

The evidence of the benefits of curriculum-based comprehensive sexuality education is strong. The most successful sexuality education programmes provide accurate and age-appropriate information and in addition, develop life skills and provide support to deal with thoughts, feelings and experiences that accompany sexual maturity (e.g. falling in love and refusing unwanted sex). They are also linked to contraceptive provision and services [15].

Although policies requiring sexuality education for adolescents are in place in many countries, they are poorly implemented, if at all. Health and education policy makers and managers must ensure that curriculum-based sexuality education is widely and effectively implemented. Complementary efforts are needed to reach the many adolescents who are not in school [1].

Because many adolescents have knowledge gaps and misconceptions about contraception and their side effects, they must be provided accurate information and given opportunities to ask questions and discuss their concerns. They must also be told where they could get contraception [1].

#### Improving access to contraception

This means making a wide range of contraceptive methods available and accessible to adolescents, and supporting them to choose a methods that meet their special needs through counselling. In line with WHO’s eligibility criteria on contraceptive provision [16], a range of methods are appropriate for adolescents as age alone is not a contraindication for any method (apart from sterilization). Long acting reversible methods such as intrauterine devices or implants can also be good choices for adolescents depending on their needs and preferences.

Adolescents in many places are unwilling to visit facilities providing contraception because they view them as unfriendly. There is growing evidence of the value of making health services adolescent friendly [16]. WHO’s Guidelines on adolescent pregnancy call for making health services adolescent friendly to make it easier for adolescents to obtain the contraceptive methods they need [1].

What are Adolescent Friendly Health Services?

To be considered adolescent-friendly, health services should be accessible, acceptable, equitable, appropriate and effective, as outlined below [16]:

Accessible

Adolescents *are able to* obtain the health services that are available

Acceptable

Adolescents *are willing to* obtain the health services that are available

Equitable

*All adolescents, not just some groups of adolescents*, are able to obtain the health services that are available

Appropriate

The *right health services (i.e. the ones they need)* are provided to them

Effective

The *right health services are provided in the right way*, and make a positive contribution to their health

To improve access to contraception, health facilities must be made easy to get to and welcoming, they must have adequate stocks of a range of contraceptive methods, and adolescents must be supported to choose the ones that meet their needs and preferences by empathetic and competent health workers.

Contraceptive education, counselling and provision could be integrated into other health services used by adolescents – including STI management, HIV counselling and testing, comprehensive abortion care services and postpartum care. For many adolescents, contact with these services may be their first opportunity to have a face-to-face discussion about contraception with a competent person. Integration into postpartum services offers the opportunity to reach first-time mothers with information on birth spacing so they can delay a second pregnancy.

In making health services adolescent friendly, it is important to build on what already exists - modifying general health facilities and building the competencies and attitudes of existing health-service providers, rather than setting up new facilities and assigning some health-service providers exclusively for adolescents. Having said this, dedicated health facilities could be useful to reach marginalized groups of adolescents (such as sex workers) who may be reluctant to use a service-delivery point open to all [17].

Even if health facilities are adolescent-friendly, they are unlikely to attract all adolescents [18]. Therefore, contraception should be provided through a variety of outlets. Outreach to adolescents in venues where they socialize can improve their access to contraceptive information and services – on the spot or through referral [19]. Making pharmacies and shops adolescent-friendly could greatly expand ready access to over-the-counter contraceptive methods. Some countries have begun to task-shift contraceptive services to community-level providers in response to shortages of qualified medical personnel [20]. Adolescents could benefit from these efforts if confidentiality can be assured.

In summary, there is fairly good evidence - from research studies and small-scale and time limited projects – on effective ways of increasing access and use of contraception by adolescents. They include favourable laws and policies; multifaceted communication programmes directed at community leaders and members, and at adolescents - that inform, educate and create supportive norms for the provision and use of contraception; accurate and age-appropriate curriculum based sexuality education; and the provision of a wide range of contraceptive methods through different adolescent-friendly outlets [1]. The challenge is to build on these small-scale and time-limited initiatives to build large scale and sustained programmes [21]*.*

## Conclusion

Substantial numbers of adolescents, both married and unmarried are at risk of pregnancy with serious health and social costs to young mothers and their babies. Poor access to and use of contraception is a key contributory factor. From research studies and projects there is compelling evidence of effective interventions to improve access to and use of contraceptive information and services to different groups of adolescents in a variety of resource-constrained settings.

To meet the needs and fullfil the rights of adolescents, countries should eliminate medical and social restrictions to the provision of contraception to adolescents, and support and enable adolescents to obtain contraceptive methods that are appropriate to their needs and preferences through delivery mechanisms that are acceptable to them.

## Appendix

Reviews and studies which fed into the development of WHO’s Guidelines on preventing early pregnancy and poor reproductive outcomes in adolescents in developing countries.

1. Oringanje C, Meremikwu MM, Eko H, et al. Interventions for preventing unintended pregnancies among adolescents. Cochrane Database of Systematic Reviews; 2009.

2. Lopez LM, Hiller JE, Grimes DA. Education for contraceptive use by women after childbirth. Cochrane Database of Systematic Reviews; 2010.

3. Chen X, Lunn S, Deveaux L, et al. A cluster randomized controlled trial of an adolescent HIV prevention program among Bahamian youth: Effect at 12 months post-intervention. AIDS Behav 2009;13:499e508.

4. Kinsler J, Sneed CD, Morisky DE, Ang A. Evaluation of a school-based intervention for HIV/AIDS prevention among Belizean adolescents. Health Educ Res 2004;19:730e8.

5. Andrade H, Mello M, Sousa M, et al. Changes in sexual behavior following a sex education program in Brazilian public schools. Cadernos de Saúde Pública 2009;25:1168e76.

6. Magnani R, Gaffkin L, de Aquino EM, et al. Impact of an integrated adolescent reproductive health program in Brazil. Stud Fam Plan 2001;32:230e43.

7. Pulerwitz J, Barker G. Promoting healthy relationships and HIV/STI prevention for young men: Positive findings from an intervention study in Brazil. New York: Population Council; 2004.

8. Van Rossem R, Meekers D. An evaluation of the effectiveness of targeted social marketing to promote adolescent and young adult reproductive health in Cameroon. AIDS Educ Prevent 2000;12:383e404.

9. Plautz A, Meekers D. Evaluation of the reach and impact of the 100% Jeune youth social marketing program in Cameroon: Findings from three crosssectional surveys. Reproduct Health 2007;4:1. http://dx.doi.org/10.1186/1742-4755-4-1.

10. Speizer I, Tambashe B, Tegang S. An evaluation of the “Entre Nous Jeunes” peer-educator program for adolescents in Cameroon. Stud Fam Plan 2001; 32:339e51.

11. Meekers D, Agha S, Klein M. The impact on condom use of the “100% Jeune” social marketing program in Cameroon. J Adolesc Health 2005;36:530. e1e530.e12

12. Murray N, et al. An evaluation of an integrated adolescent development program for urban teenagers in Santiago, Chile. March 5, 2000 (unpublished).

13. Villarruel A, et al. Examining the long term effects of Cuidate: A sexual risk reduction program in Chile. Revista Panamericana de Salud Pública 2010; 27:345e51.

14. Wang B, Hertog S, Meier A, et al. The potential of comprehensive sex education in China: Findings from suburban Shanghai. Intl Fam Plan Perspect 2005;31:63e72.

15. Lou C, Wang B, Shen Y, Gao ES. Effects of a community-based sex education and reproductive health service program on contraceptive use of unmarried youths in Shanghai. J Adolesc Health 2004;34:433e40.

16. Tu X, Lou C, Gao E, Shah IH. Long-term effects of a community-based program on contraceptive use among sexually active unmarried youth in Shanghai, China. J Adolesc Health 2008;42:249e58.

17. Daniel E, Masilamani R, Rahman M. The effect of community-based reproductive health communication interventions on contraceptive use among young married couples in Bihar, India. Intl Fam Plan Perspect 2008; 34:189e97.

18. Erulkar A, Ettyang LI, Onoka C, et al. Behavior change evaluation of a culturally consistent reproductive health program for young Kenyans. Intl Fam Plan Perspect 2004;30:58e67.

19. Maticka-Tyndale E, Wildish J, Gichuru M. Quasi-experimental evaluation of a national primary school HIV intervention in Kenya. Eval Program Plan 2007;30:172e86.

20. Nuekom J, Ashford L. Changing youth behavior through social marketing. Program experiences and research findings from Cameroon, Madagascar, and Rwanda. Washington DC: Population Reference Bureau; 2003.

21. Center For Development and Population Activities (CEDPA). Reproductive health for youth in Mali Project (RHYM): End of project report. 2003 (unpublished).

22. Gallegos E, et al. Intervención para reducir riesgo en conductas sexuales de adolescentes: Un ensayo aleatorizado y controlado. Salud Pública de México 2008;50:59e66.

23. Center for Research on Environment, Health, and Population Activities (CREHPA). Determining an effective and replicable communication-based mechanism for improving young couples’ access to and use of reproductive health information and services in Nepal: An operations research study. Kathmandu, Nepal: Center for Research on Environment, Health and Population Activities (CREHPA); 2004.

24. Muewissen LE, Gorter AC, Knottnernus AJA. Impact of accessible sexual and reproductive health care on poor and underserved adolescents in Managua, Nicaragua: A quasi-experimental intervention study. J Adolesc Health 2006;38:56.e1e56.e9.

25. Casey S, Larsen MM, McGinn T, et al. Changes in HIV/AIDS/STI knowledge, attitudes, and behaviours among the youth in Port Loko, Sierra Leone. Glob Public Health 2006;1:249e63.

26. Harvey B, Stuart J, Swan T. Evaluation of a drama-in-education programme to increase AIDS awareness in South African high schools: A randomized community intervention trial. Int J Sex Transmit Dis AIDS 2000;11:105e11.

27. James S, Reddy P, Ruiter RA, et al. The impact of an HIV and AIDS life skills program on secondary school students in Kwazulu-Natal, South Africa. AIDS Educ Prev 2006;18:281e94.

28. Doyle A, Ross DA, Maganja K, et al. Long-term biological and behavioural impact of an adolescent sexual heath in intervention in Tanzania: Follow up survey of community-based MEMA Kwa Vijana trial. Plos Med 2010;7:e1000287.

29. Fitzgerald A, Stanton BF, Terreri N, et al. Use of western-based HIV risk reduction interventions targeting adolescents in an African setting. J Adolesc Health 1999;25:52e61.

30. Thato R, Jenkins RA, Dusitsin N. Effects of the culturally-sensitive comprehensive sex education programme among Thai secondary school students. J Adv Nurs 2008;62:457e69.

31. Askew I, Chege J, Njue C, Radeny S; Kenya Ministry of Health, Ministry of Education, Science and Technology, Ministry of Gender, Sport, Culture and Social Services. A multi-sectoral approach to providing reproductive health information and services to young people in western Kenya: The Kenya Adolescent Reproductive Health Project. New York: The Population Council; 2004.

32. Sant’Anna M, Carvalho KA, Melhad A, et al. Teenage pregnancy: Impact of the integral attention given to the pregnant teenager and adolescent mother as a protective factor for repeat pregnancy. Scientific World J 2007;7:187e94.

33. Brieger W, Delango GE, Lance CG, et al. West African Youth Initiative: Outcome of a reproductive health education program. J Adolesc Health 2001;29:439e46.

34. Ross DA, Changalucha J, Obasi AIN, et al. Biological and behavioural impact of an adolescent sexual health intervention in Tanzania: A community randomized trial. AIDS 2007;21:1943e55.

## Abbreviations

LMIC: Low and middle income countries; DHS: Demographic and Health Surveys; WHO: World Health Organization; EC: Emergency contraception; STI: Sexually Transmitted Infection; HIV: Human Immuno-Deficiency Virus.

## Competing interests

The authors declare that they have no competing interest.

## Authors’ contributions

C-M conceived the paper. W prepared the first draft of the paper. C-M, W, MC and P prepared the draft submitted to Reproductive Health. H was one of the reviewers. The set of four initial authors invited her to coauthor the paper, in appreciation of her detailed and helpful comments on the draft that had been submitted. All five authors contributed to finalizing the paper. All authors read and approved the final manuscript.

## Authors’ information

Chandra-Mouli and Phillips work for the World Health Organization. McCarraher works for FHI360 and Williamson previously worked there. She is now with the University of North Carolina. Hainsworth works for Pathfinder International.
